# True Bravery

**Published:** 1878-10-20

**Authors:** 


**True Bravery.**—A few weeks ago the Medico-Chirurgical Society of Atlanta, appointed a committee of three of its members to visit the city of Chattanooga for the purpose of examining the fever prevailing at that point, and to report to the society their views of its true character. The committee appointed consisted of Drs. J. J. Knott, A. R. Alley, and Dr. H. B. Lee.

They accordingly visited the city, and conferred with the medical gentlemen of the place, who kindly and courteously exhibited their cases to the committee.

Their observations resulted in the decision that the disease was genuine yellow fever, which they reported upon their return, and subsequently tendered their services, if needed, to the afflicted city.

In a few days the fever cases having increased the call for help was made, and was promptly responded to by Drs. Knott, Lee and Olmstead, who went to the afflicted city, established a hospital, called the *Atlanta Hospital*, and supported by the sympathizing citizens of Atlanta, and which is doing successful and noble work in behalf of many yellow fever sufferers.

These brethren of the profession and members of the Medico-chirugical Society have voluntarily engaged in this noble and philanthropic work, and they should have an appreciative commendation, so also all those resident practitioners who have bravely stood at their post and battled with the scourge. As mind is infinitly superior to matter, so is moral heroism to mere physical courage. The demonstrations of the one can elicit shouts of applause from the rabble, but the other is a sublimated essence of the soul approaching the divine, and which angelic spectators look down upon with glad approval. These brethren like many others during this fearful trial of our sorely scourged Southern cities, have with true bravery stepped out from the almost impregnable fortress of a healthful locality to face the deadly missiles of the epidemic that are falling thick and fast upon the flower-scented breezes of these potions of our southern land. Instead of a shower of bullets from an enemy's rank drawn up in position on the battle field, they are in the midst of a rain of death more fearful from its being invisible in its descent upon its victims; who per force, are powerless to elude its noiseless and insidious approaches.

We trust that a kind Providence will bless the noble and heroic action of these Brethren who have gone from our city and other cities; that they will be shielded from the fatal darts of the plague, and under God be eminently successful in restoring many of the afflicted sufferers to health and to their friends.

If upon the fall of the last victim of this epidemic an angelic messenger should visit the earth and, hovering over our stricken cities, call the roll of the heroes who had bravely fought this fearful scourge since its first appearance many names that men "would not willingly let die though their owners were still in life might respond to the summons."

But the grand self immolated host lying in the dreamless sleep that has no waking, who could answer to their names? Who but the heavenly recorder of men's good deeds and earth's martyred legions. Their names can never be trumpeted to earth by fame, but they are written upon the celestial arches, and when the roll-call of the Eternal City resounds within its jeweled walls no voice will then respond "unknown."

